# Coronary Artery Bypass Graft Surgery: The Past, Present, and Future of Myocardial Revascularisation

**DOI:** 10.1155/2014/726158

**Published:** 2014-01-02

**Authors:** Michael Diodato, Edgar G. Chedrawy

**Affiliations:** ^1^University of Connecticut, Hartford Hospital, Division of Surgical Critical Care, 80 Seymour Street, Hartford, CT 06102-5037, USA; ^2^University of Illinois at Chicago and Medical Director, Vanguard Weiss Memorial Hospital, 4646 N Marine Drive, Suite 7C, Chicago, IL 60640, USA

## Abstract

The development of the heart-lung machine ushered in the era of modern cardiac surgery. Coronary artery bypass graft surgery (CABG) remains the most common operation performed by cardiac surgeons today. From its infancy in the 1950s till today, CABG has undergone many developments both technically and clinically. Improvements in intraoperative technique and perioperative care have led to CABG being offered to a more broad patient profile with less complications and adverse events. Our review outlines the rich history and promising future of myocardial revascularization.

## 1. History

Coronary artery bypass grafting (CABG) is defined as “open-heart surgery in which a section of a blood vessel is grafted from the aorta to the coronary artery to bypass the blocked section of the coronary artery and improve the blood supply to the heart.” The pathophysiology of coronary artery disease was established in 1876 by Adam Hammer when he postulated that angina (imbalance of coronary perfusion supply and demand) was caused by interruption of coronary blood supply and that myocardial infarction occurred after the occlusion of at least one coronary artery [1]. In the 19th century heart surgery was performed infrequently and with poor results. In 1896, Stephen Paget wrote that “surgery of the heart has probably reached the limits set by nature to all surgery” [2]. In that same year, Ludwig Rehn successfully conducted heart surgery repairing a stab wound [3]. In 1910, Alexis Carrel was the first to describe CABG [4].

Cardiac surgery became more feasible in the late 1930s with the development of the heart-lung machine by Dr. John Gibbon which enabled cardiopulmonary bypass (CPB) [5]. In 1950, at McGill University in Montreal, QC, Canada, Vineburg and Buller were the first to implant the internal mammary artery (IMA) into the myocardium to treat cardiac ischemia and angina [6]. In 1953, D. W. Gordon Murray reported placement of arterial grafts in the coronary circulation [7]. Shortly thereafter, in 1955, Sidney Smith was the first to harvest saphenous vein and use it as a graft from aorta to into the myocardium [7]. In 1958, Longmire et al. performed the first open coronary artery endarterectomy without CPB at University of California at Los Angeles (UCLA) [8].

The 1960s saw great advances in coronary artery surgery. Goetz et al. are credited with performing the first successful human coronary artery bypass operation in 1961 [9]. In 1962, Proudfit et al. produced the first practical cardiac angiography visualizing the coronary arteries [10]. Kolesov performed the first successful internal mammary artery-coronary artery anastomosis in 1964 [11], and Favoloro et al. reported using saphenous vein to restore coronary artery blood flow in 171 patients [12]. In the 1970s, continued development of technique and conduits occurred. In 1973, Benetti, Calafiore, and Subramian successfully completed anastamoses on a beating heart [13]. In the 1980s, the prevalence of CABG increased and safety improved. Thoracoscopic harvesting of the left IMA was reported in 1998 by Duhaylongsod et al. [14], and minimally invasive and robotic surgical approaches were also developed [15, 16] Currently, the number of CABG is declining from a peak of 519,000 operations in 2000 to an estimated 300,000 cases in 2012 [17].

## 2. Methods

Although the fundamental basis of CABG is to reestablish perfusion to the myocardium, there are several different approaches to accomplish this goal. The first factor considered is the utilization of cardiopulmonary bypass or “on pump versus off pump.” Initially, most cardiac surgeries were performed on a beating heart, but with the development of cardiopulmonary bypass and cardioplegia, most CABG were performed on pump. However, interest in off-pump coronary artery bypass (OPCAB) surgery had resurgence in the 1990s. Benetti et al. [18] and Buffalo et al. [19] published their results of nearly 2000 OPCAB patients showing operative safety. Reported benefits of OPCAB include lower end organ damage, that is, renal failure, cerebrovascular accidents (CVA), fewer cognitive deficits, less psychomotor defects, lower transfusion rates, and reduced systemic inflammation [20].

Recently, Afilalo et al. published a meta-analysis comparing on-pump CABG and OPCAB [21]. The primary outcomes were all-cause mortality, stroke, and myocardial infarction. Fifty-nine trials were included with nearly 9000 patients. The study population had a mean age of 63.4 and with a male to female predominance of over 4 : 1. Postoperative CVA was significantly reduced by 30% in the OPCAB group (risk ratio (RR) 0.70, 95% CI: 0.49–0.99). Rate in mortality (RR: 0.90, 95% CI: 0.63–1.30) and myocardial infarction (pooled RR: 0.89, 95% CI: 0.69–1.13) were not different between groups. In the metaregression analysis, clinical outcome was similar regardless of mean age, proportion of females in the trial, number of grafts per patient, and trial publication date.

Forouzannia et al. compared clinical and economic outcomes of off-pump and on-pump coronary artery bypass surgery [22]. They analyzed 304 patients undergoing coronary artery bypass surgery and were randomized into conventional on pump and off-pump groups. OPCAB significantly reduced the need for postoperative transfusion requirement (*P* < 0.05). There were no statistically significant differences in surgical reexploration or length of stay. They found that the mean cost for an on-pump surgery was significantly higher than an off-pump surgery.

Interestingly, Yadava et al. reviewed 3500 patients over 8 years. 14.6% of patients were women [23]. In-hospital mortality was higher in women as compared to men, 2.92% versus 1.8%. The most common causes of mortality were low cardiac output and renal failure. Use of OPCAB reduced mortality (1.84% versus 4.5% on pump;  *P* = 0.01) in women. Blood transfusions (2.5 ± 1.2 units versus 4.3 ± 1.4; units *P* < 0.001); ICU stay (29.4 ± 16.4 h versus 38.3 ± 17.3 h;  *P* < 0.0001); and length of stay (6.81 ± 1.6 d versus 8.05 ± 2.1 d; *P* < 0.0001) were also reduced in the OPCAB female cohort.

In 2009, the results of the ROOBY (randomized on/off bypass) trial were published, reporting the outcomes for 2.203 patients (99% men) at 18 Veterans Affairs Medical Centers [24]. The primary short-term endpoint, a composite of death or complications within 30 days of surgery, occurred with similar frequency (5.6% for on-pump CABG; 7.0% for off-pump CABG; *P* = 0.19). The primary long-term endpoint, a composite of death from any cause, a repeat revascularization procedure, or a nonfatal myocardial infarction (MI) within 1 year of surgery, occurred more often in those undergoing off-pump CABG (9.9%) than in those having on-pump CABG (7.4%; *P* = 0.04). Neuropsychological outcomes were not different between the groups, and graft patency was higher in the on-pump group (87.8% versus 82.6%; *P* = 0.01) at 12 months.

Minimally invasive and robotic assisted approaches have also been developed. Minimally invasive cardiac surgery does not use CPB and can be performed through smaller incisions. This approach has gained popularity and is most often used for LIMA to LAD grafts. Additional benefits may also include reduced operative time, reduced recover time, decreased need for blood transfusion, less time under anesthesia, decreased length of ICU stay, less pain, and an estimated 40% savings over conventional CABG [25]. However, the total number of bypassable vessels is reduced secondary to exposure making these approaches useful for a select group of patients.

## 3. Conduits

Multiple conduits may be employed to establish cardiac revascularization. In the 2011 CCF/AHA Guidelines for Coronary Artery Bypass Graft Surgery advocated the use of arterial grafts for anastomosis to the LAD [26]. The LIMA is the vessel of first choice. IMAs usually are patent for many years postoperatively (10-year patency >90%) [27] because of the fact that <4% of IMAs develop atherosclerosis, and only 1% have atherosclerotic stenoses of hemodynamic significance [28].

Reversed saphenous vein grafts (SVGs) are commonly used in patients undergoing CABG. Their disadvantage is a declining patency with time: 10% to as many as 25% of them occlude within 1 year of CABG [29]; an additional 1% to 2% occlude each year during the 1 to 5 years after surgery; and 4% to 5% occlude each year between 6 and 10 years postoperatively. Therefore, 10 years after CABG, 50% to 60% of SVGs are patent, only half of which have no angiographic evidence of atherosclerosis [30].

Other arterial conduits, such as the radial, gastroepiploic, and inferior epigastric arteries, have been used in CABG. Radial artery graft patency is best when used to graft a left-sided coronary artery with high grade stenosis and worst when utilized on the lower pressure right heart. The gastroepiploic artery is most often used to bypass the right coronary artery or its branches, but it is prone to spasm [31]. The 1-, 5-, and 10-year patency rates of the gastroepiploic artery are reportedly 91%, 80%, and 62%, respectively [32]. Due to its length, the inferior epigastric artery is usually used as a “Y” or “T” graft or may be used as free graft. It is also prone to spasm. Its reported 1-year patency is about 90% [33].

## 4. CABG versus Stenting

In April 2012, the results of the ASCERT trial were published in the New England Journal of Medicine. This was a study combining databases of the ACCF National Cardiovascular Data Registry and the STS Adult Cardiac Surgery Database to claims data from the Centers for Medicare and Medicaid Services for the years 2004 through 2008 [34]. The study reviewed the records of nearly 190.000 patients 65 years or older with two- or three-vessel disease. 86.244 underwent CABG and 103.549 underwent PCI. The median follow-up period was 2.67 years. At 1 year, there was no significant difference in mortality between the groups (6.24% CABG versus 6.55% PCI; risk ratio, 0.95). At 4 years, there was lower mortality with CABG than with PCI (16.4% versus 20.8%; risk ratio, 0.79).

A systematic review of the 22 RCTs comparing CABG with balloon angioplasty or stent implantation was performed [35]. The authors concluded that survival was similar for CABG and PCI at 1 and 5 years. Survival was the same for single and multivessel CAD. The incidence of MI was similar at 5 years after randomization. CVA occurred more commonly with CABG than with PCI (1.2% versus 0.6%). Relief of angina was more frequently improved with CABG than with PCI at 1 and 5 years. Repeat coronary revascularization was required less after CABG than after PCI at both 1 year (3.8% versus 26.5%) and 5 years of followup (9.8% versus 46.1%).

## 5. Common Adverse Events

The incidence of postoperative CVA after CABG ranges from 1.4% to 3.8% [36]. Risk factors include age, previous stroke, diabetes mellitus, hypertension [37], and female sex [38]. Hypoperfusion is also risk factor for postoperative stroke [39]. Mortality rate is 10-fold higher among post-CABG patients with prior stroke with longer lengths of hospital stay [40]. Although off-pump CABG was introduced to reduce adverse neurological outcomes associated with CPB, this has not been proven in the literature. The incidence of postoperative delirium after CABG is <10% [41]. Postoperative delirium has been linked to functional decline at 1 month, short-term cognitive decline, and risk of late mortality [42]. Short-term cognitive changes occur in some patients after on-pump CABG. Risk factors for short-term postoperative cognitive decline include preexisting risk cerebrovascular disease, central nervous system disorders, and cognitive impairment [43–45]. It is believed that nearly 30% of CABG patients may have preoperative cognitive impairment.

Nosocomial infections occur in 10% to 20% of cardiac surgery patients. To prevent surgical site infections in CABG patients, a multimodality approach involving several perioperative interventions must be considered. Risk of deep sternal wound infection is increased in diabetics, obese patients (body mass index >30 kg/m^2^), and patients with COPD and has also been associated with prolonged CPB time, prolonged intubation time, and surgical reexploration [46–49].Infection rates may be improved by smoking cessation, optimizing nutritional status, tight glucose control, and weight loss.

Transfusion of homologous blood has been correlated, in a dose-dependent manner, to an increased risk of postoperative infection, morbidity, and both early and late death [50]. They have been additionally associated with a higher incidence of sternal wound infections [51]. In a retrospective analysis of 15.592 cardiovascular patients, the risk of sepsis and sternal wound infections increased with each unit of blood transfused [52]. This finding correlates with a RCT showing that leukocyte-depleted blood had reduced rates of infection (17.9% versus 23.5%; *P* < 0.04) and 60-day mortality (7.8% versus 3.6%; *P* < 0.019) [53]. Transfusions have also been identified as an independent risk factor for adverse outcomes [54]. Commonly, postoperative myocardial depression is observed consistently after transfusion in a dose-dependent manner. Survival rates after CABG are reduced in patients requiring transfusion [55].

The reported incidence of acute renal failure (ARF) after CABG is 2% to 3% with 1% of those patients requiring dialysis [56]. There are multiple conditions that influence postoperative renal failure. These risk factors include pre-existing renal dysfunction, decreased cardiac output, as in CHF or shock, insulin dependent diabetes, and concomitant peripheral artery disease. Advanced age, black race, female gender, and the need for emergent surgical intervention or preoperative intraaortic balloon support have all been implicated in increasing the risk of ARF [57–60].

Post-CABG myocardial dysfunction is another commonly seen adverse event. Intraaortic balloon counterpulsation has been shown to increase cardiac output and to improve coronary blood flow [58]. Several studies have shown that patients with a left ventricular ejection fraction of <30% or with left main disease have a mortality benefit with the perioperative use of an IABP. The PREVENT IV trial suggests that cardiac serum biomarkers for myonecrosis are elevated postoperatively even in roughly 10% of CABG subjects. Furthermore, both the short (30-day) and long-term (2-year) outcomes were worse in these patients, and this correlated with the degree of biomarker elevation [61].

Postoperative atrial fibrillation (AF) is the most common post-CABG adverse events and occurs in 20% to 50% of patients. Mariscalco et al. published an observational study of 1.878 consecutive subjects undergoing CABG. They noted that post-CABG AF was associated with a 4-fold increased risk of disabling CVA and 3-fold increased risk of cardiac-related death [62]. There are multiple conditions which predispose patients to postoperative AF. These include the presence of peripheral artery disease, COPD, concomitant valvular heart disease, previous cardiac surgery, preoperative AF, and pericarditis. Male gender and advanced age are also risk factors for AF. Postoperative AF almost always occurs within 5 days of surgery peaking on postoperative day 2 [63]. Multiple pharmacologic interventions have been attempted, but only perioperative beta blockade and amiodarone have been shown to be effective in reducing AF [64]. Isolated post-CABG AF usually resolves spontaneously within 6 weeks of surgery. As such, rate control with beta blockers or conversion with amiodarone is the first line of treatment [65]. Postoperative anticoagulation may be warranted in rate controlled patients still in fibrillation.

## 6. Future Directions

Advances in medical therapy and percutaneous intervention have led to ever shrinking numbers of CABG being performed each year. Furthermore, the patients undergoing these procedures have a much more complicated combination of disease processes. The future of coronary artery bypass grafting is making these difficult procedures better tolerated by this complex subset of patients through smaller incisions or without any incision.

Operative changes and challenges are trying to be addressed. Minimally invasive procedures and approaches will continue to be developed. Robotic intervention strives for a totally endoscopic CABG. Anastomotic devices are being researched to make this goal more feasible. However, most of these devices are infrequently utilized and are in the infancy of their potential development [66]. Additionally, many of the patients have extensive coronary artery disease with prior attempts at revascularization. The determination of graft patency, intraoperatively, in these patients is vital. For this reason, several techniques using transit-time flow and intraoperative fluorescence imaging are being developed. However, neither method has been proven to be adequate in the assessment of small abnormalities in graft patency [67].

The development of “hybrid suites” that allows for simultaneous or staged CABG and stenting procedures is currently being explored. These procedures combine grafting the LAD with the LIMA and stenting of the non-LAD arteries. This has been proposed to decrease the morbidity rate of traditional CABG in high-risk patients. The National Institutes of Health has sponsored a randomized control trial to evaluate the hybrid procedure versus CABG or stenting alone [66]. Additionally, nonoperative placement of substances known to promote myocardial regeneration and angiogenesis is being researched [68, 69]. With the success of stem cell therapy and molecular medicine in other fields of science and medicine, this has great potential for myocardial repair.

## 7. Summary

In a little over a century, heart surgery has gone from prohibitive to commonplace. Major advances have made the CABG a much safer and more accepted procedure. Continued research into different approaches, methods and medical interventions may make cardiac surgery less invasive and safer in the future. The benefits and risks for each patient must be evaluated with a team approach to determine which method is best for that patient. Even with paradigm shifts in medical treatments and stenting, the continued development of coronary surgery is vital for those patients who cannot be managed nonsurgically. As surgical interventions become relatively less common, the issue of how many and how to train future cardiac surgeons may become an issue. Furthermore, as the procedures and patients become more complex, the development of different specialized postoperative strategies will need to be considered. Lastly, the field of cardiac surgery will need to become more specialized as people are surviving cardiac operations for longer period of time and may need further interventions such as higher risk reinterventions.

## References

[1] Westaby S (1997). *Landmarks in Cardiac Surgery*.

[2] Davies MK, Hollman A (2002). History of cardiac surgery. *Heart*.

[3] http://en.wikipedia.org/wiki/Cardiac_surgery.

[4] Shumacker HB (1992). *The Evolution of Cardiac Surgery*.

[5] Cooper DKC (2010). *Open Heart: The Radical Surgeons Who Revolutionized Medicine*.

[6] Shrager B (1994). The vineberg procedure: the immediate forerunner of coronary artery bypass grafting. *Annals of Thoracic Surgery*.

[7] http://biomed.brown.edu/Courses/BI108/BI108_2004_Groups/Group03/History.htm.

[8] Longmire WP, Cannon J, Kattus AA (1958). Direct-vision coronary endarterectomy for angina pectoris. *The New England Journal of Medicine*.

[9] Goetz RH, Rohman M, Haller JD, Dee R, Rosnak SS (1961). Internal mammary-coronary artery anastomosis. A nonsuture method employing tantalum rings. *The Journal of Thoracic and Cardiovascular Surgery*.

[10] Proudfit WL, Shirey EK, Sones FM (1966). Selective cine coronary arteriography. Correlation with clinical findings in 1,000 patients. *Circulation*.

[11] Olearchyk AS (1988). Vasilii I. Kolesov: a pioneer of coronary revascularization by internal mammary-coronary artery grafting. *Journal of Thoracic and Cardiovascular Surgery*.

[12] Captur G (2004). Memento for René Favaloro. *Texas Heart Institute Journal*.

[13] Cremer J, Fraund S (2004). *Beating Heart Bypass Surgery and Minimally Invasive Conduit Harvesting*.

[14] Duhaylongsod FG, Mayfield WR, Wolf RK (1998). Thoracoscopic harvest of the internal thoracic artery: a multicenter experience in 218 cases. *Annals of Thoracic Surgery*.

[15] Falk V, Diegler A, Walther T, Autschbach R, Mohr FW (2000). Developments in robotic cardiac surgery. *Current Opinion in Cardiology*.

[16] Prasad SM, Ducko CT, Stephenson ER, Chambers CE, Damiano RJ (2001). Prospective clinical trial of robotically assisted endoscopic coronary grafting with 1-year follow-up. *Annals of Surgery*.

[17] Gaziano T, Reddy KS, Paccaud F, Horton S, Chaturvedi V, Jamison DT, Breman JG, Measham AR (2006). Cardiovascular disease. *Disease Control Priorities in Developing Countries*.

[18] Benetti FJ, Naselli G, Wood M, Geffner L (1991). Direct myocardial revascularization without extracorporeal circulation; Experience in 700 patients. *Chest*.

[19] Buffalo E, de Andrade JCS, Branco JNR, Teles CA, Aguiar LF, Gomes WJ (1996). Coronary artery bypass grafting without cardiopulmonary bypass. *Annals of Thoracic Surgery*.

[20] Møller CH, Penninga L, Wetterslev J, Steinbrüchel DA, Gluud C (2012). Off-pump versus on-pump coronary artery bypass grafting for ischaemic heart disease. *Cochrane Database of Systematic Reviews*.

[21] Afilalo J, Rasti M, Ohayon SM, Shimony A, Eisenberg MJ (2012). Off-pump versus on-pump coronary artery bypass surgery: an updated meta-analysis and meta-regression of randomized trials. *European Heart Journal*.

[22] Forouzannia SK, Abdollahi MH, Mirhosseini SJ (2011). Clinical outcome and cost in patients with off-pump versus on-pump coronary artery bypass surgery. *Acta Medica Iranica*.

[23] Yadava OP, Prakash A, Kundu A, Yadava M (2011). Coronary artery bypass grafting in women—is OPCAB mandatory?. *Indian Heart Journal*.

[24] Shroyer AL, Grover FL, Hattler B (2009). On-pump versus off-pump coronary-artery bypass surgery. *The New England Journal of Medicine*.

[25] Mack M, Acuff T, Yong P, Jett GK, Carter D (1997). Minimally invasive thoracoscopically assisted coronary artery bypass surgery. *European Journal of Cardio-Thoracic Surgery*.

[26] Cameron A, Davis KB, Green G, Schaff HV (1996). Coronary bypass surgery with internal-thoracic-artery grafts: effects on survival over a 15-year period. *The New England Journal of Medicine*.

[27] Fiore AC, Naunheim KS, Dean P (1990). Results of internal thoracic artery grafting over 15 years: single versus double grafts. *Annals of Thoracic Surgery*.

[28] Sims FH (1983). A comparison of coronary and internal mammary arteries and implications of the results in the etiology of arteriosclerosis. *American Heart Journal*.

[29] FitzGibbon GM, Kafka HP, Leach AJ, Keon WJ, Hooper GD, Burton JR (1996). Coronary bypass graft fate and patient outcome: angiographic follow-up of 5,065 grafts related to survival and reoperation in 1,388 patients during 25 years. *Journal of the American College of Cardiology*.

[30] Bourassa MG, Fisher LD, Campeau L, Gillespie MJ, McConney M, Lespérance J (1985). Long-term fate of bypass grafts: the Coronary Artery Surgery Study (CASS) and Montreal heart institute experiences. *Circulation*.

[31] Glineur D, D’hoore W, El Khoury G (2008). Angiographic predictors of 6-month patency of bypass grafts implanted to the right coronary artery. A prospective randomized comparison of gastroepiploic artery and saphenous vein grafts. *Journal of the American College of Cardiology*.

[32] Suma H, Isomura T, Horii T, Sato T (2000). Late angiographic result of using the right gastroepiploic artery as a graft. *Journal of Thoracic and Cardiovascular Surgery*.

[33] Buche M, Schroeder E, Gurné O (1995). Coronary artery bypass grafting with the inferior epigastric artery. Midterm clinical and angiographic results. *Journal of Thoracic and Cardiovascular Surgery*.

[34] Weintraub WS, Grau-Sepulveda MV, Weiss JM (2012). Comparative effectiveness of revascularization strategies. *The New England Journal of Medicine*.

[35] Bravata DM, Gienger AL, McDonald KM (2007). Systematic review: the comparative effectiveness of percutaneous coronary interventions and coronary artery bypass graft surgery. *Annals of Internal Medicine*.

[36] Selim M (2007). Perioperative stroke. *The New England Journal of Medicine*.

[37] McKhann GM, Goldsborough MA, Borowicz LM (1997). Predictors of stroke risk in coronary artery bypass patients. *Annals of Thoracic Surgery*.

[38] Filsoufi F, Rahmanian PB, Castillo JG, Bronster D, Adams DH (2008). Incidence, topography, predictors and long-term survival after stroke in patients undergoing coronary artery bypass grafting. *Annals of Thoracic Surgery*.

[39] Gottesman RF, Sherman PM, Grega MA (2006). Watershed strokes after cardiac surgery: diagnosis, etiology, and outcome. *Stroke*.

[40] Roach GW, Kanchuger M, Mangano CM (1996). Multicenter study of perioperative ischemia research group and the ischemia research and education foundation investigators. Adverse cerebral outcomes after coronary bypass surgery. *The New England Journal of Medicine*.

[41] Légaré JF, Buth KJ, King S (2004). Coronary bypass surgery performed off pump does not result in lower in-hospital morbidity than coronary artery bypass grafting performed on pump. *Circulation*.

[42] Rudolph JL, Inouye SK, Jones RN (2010). Delirium: an independent predictor of functional decline after cardiac surgery. *Journal of the American Geriatrics Society*.

[43] Ho PM, Arciniegas DB, Grigsby J (2004). Predictors of cognitive decline following coronary artery bypass graft surgery. *Annals of Thoracic Surgery*.

[44] Goto T, Baba T, Honma K (2001). Magnetic resonance imaging findings and postoperative neurologic dysfunction in elderly patients undergoing coronary artery bypass grafting. *Annals of Thoracic Surgery*.

[45] Jensen BO, Rasmussen LS, Steinbruchel DA (2008). Cognitive outcomes in elderly high-risk patients 1 year after off-pump versus on-pump coronary artery bypass grafting. A randomized trial. *European Journal of Cardio-Thoracic Surgery*.

[46] Lowenstein E, Hallowell P, Levine FH, Daggett WM, Austen WG, Laver MB (1969). Cardiovascular response to large doses of intravenous morphine in man. *The New England Journal of Medicine*.

[47] Braxton JH, Marrin CA, McGrath PD (2004). 10-Year follow-up of patients with and without mediastinitis. *Seminars in Thoracic and Cardiovascular Surgery*.

[48] Alam M, Siddiqui S, Lee V (2011). Isolated coronary artery bypass grafting in obese individuals: a propensity matched analysis of outcomes. *Circulation Journal*.

[49] Milano CA, Kesler K, Archibald N, Sexton DJ, Jones RH (1995). Mediastinitis after coronary artery bypass graft surgery: risk factors and long-term survival. *Circulation*.

[50] Leal-Noval SR, Rincón-Ferrari MD, García-Curiel A (2001). Transfusion of blood components and postoperative infection in patients undergoing cardiac surgery. *Chest*.

[51] Blanchard A, Hurni M, Ruchat P, Stumpe F, Fischer A, Sadeghi H (1995). Incidence of deep and superficial sternal infection after open heart surgery. A ten years retrospective study from 1981 to 1991. *European Journal of Cardio-Thoracic Surgery*.

[52] Banbury MK, Brizzio ME, Rajeswaran J, Lytle BW, Blackstone EH (2006). Transfusion increases the risk of postoperative infection after cardiovascular surgery. *Journal of the American College of Surgeons*.

[53] van de Watering LM, Hermans J, Houbiers JG (1998). Beneficial effects of leukocyte depletion of transfused blood on postoperative complications in patients undergoing cardiac surgery: a randomized clinical trial. *Circulation*.

[54] van Straten AH, Kats S, Bekker MW (2010). Risk factors for red blood cell transfusion after coronary artery bypass graft surgery. *Journal of Cardiothoracic and Vascular Anesthesia*.

[55] Surgenor SD, DeFoe GR, Fillinger MP (2006). Intraoperative red blood cell transfusion during coronary artery bypass graft surgery increases the risk of postoperative low-output heart failure. *Circulation*.

[56] Abraham VS, Swain JA, Gravlee GP, Davis R (2000). Cardiopulmonary bypass and kidney. *Cardiopulmonary Bypass: Principles and Practice*.

[57] Mangano CM, Diamondstone LS, Ramsay JG, Aggarwal A, Herskowitz A, Mangano DT (1998). Renal dysfunction after myocardial revascularization: risk factors, adverse outcomes, and hospital resource utilization. *Annals of Internal Medicine*.

[58] Andersson LG, Ekroth R, Bratteby LE, Hallhagen S, Wésslen O (1993). Acute renal failure after coronary surgery—a study of incidence and risk factors in 2009 consecutive patients. *Thoracic and Cardiovascular Surgeon*.

[59] Zanardo G, Michielon P, Paccagnella A (1994). Acute renal failure in the patient undergoing cardiac operation: prevalence, mortality rate, and main risk factors. *Journal of Thoracic and Cardiovascular Surgery*.

[60] Thakar CV, Arrigain S, Worley S, Yared JP, Paganini EP (2005). A clinical score to predict acute renal failure after cardiac surgery. *Journal of the American Society of Nephrology*.

[61] Alexander JH, Ferguson TB, Joseph DM (2005). The project of ex-vivo vein graft engineering via transfection IV (PREVENT IV) trial: study rationale, design, and baseline patient characteristics. *American Heart Journal*.

[62] Mariscalco G, Klersy C, Zanobini M (2008). Atrial fibrillation after isolated coronary surgery affects late survival. *Circulation*.

[63] Fuster V, Rydén LE, Cannom DS (2011). 2011 ACCF/AHA/HRS focused updates incorporated into the ACC/AHA/ESC 2006 Guidelines for the management of patients with atrial fibrillation: a report of the American College of Cardiology Foundation/American Heart Association Task Force on Practice Guidelines developed in partnership with the European Society of Cardiology and in collaboration with the European Heart Rhythm Association and the Heart Rhythm Society. *Journal of the American College of Cardiology*.

[64] Ferguson BT, Coombs LP, Peterson ED (2002). Preoperative *β*-blocker use and mortality and morbidity following CABG surgery in North America. *Journal of the American Medical Association*.

[65] Halonen J, Hakala T, Auvinen T (2006). Intravenous administration of metoprolol is more effective than oral administration in the prevention of atrial fibrillation after cardiac surgery. *Circulation*.

[66] Balacumaraswami L, Taggart DP (2007). Intraoperative imaging techniques to assess coronary artery bypass graft patency. *Annals of Thoracic Surgery*.

[67] Alexander JH, Hafley G, Harrington RA (2005). Efficacy and safety of edifoligide, an E2F transcription factor decoy, for prevention of vein graft failure following coronary artery bypass graft surgery: PREVENT IV: a randomized controlled trial. *Journal of the American Medical Association*.

[68] Chedrawy EG, Chiu RC (2002). Cellular cardiomyoplasty: cell therapy for myocardial regeneration. *Artificial Cells, Blood Substitutes, and Immobilization Biotechnology*.

[69] Simons M, Laham RJ, Post M, Sellke FW (2000). Therapeutic angiogenesis: potential role of basic fibroblast growth factor in patients with severe ischaemic heart disease. *BioDrugs*.

